# An eye-movement exploration into return-sweep targeting during reading

**DOI:** 10.3758/s13414-019-01742-3

**Published:** 2019-06-04

**Authors:** Timothy J. Slattery, Martin R. Vasilev

**Affiliations:** Department of Psychology, Bournemouth University, P104c Poole house, Talbot Campus, Fern Barrow, Poole, BH12 5BB UK

**Keywords:** Eye-movements, Reading, Return-sweep, Bold text, Saccade targeting

## Abstract

Return-sweeps are an essential eye-movement that takes the readers’ eyes from the end of one line of text to the start of the next. While return-sweeps are common during normal reading, the eye-movement literature is dominated by single-line reading studies where no return-sweeps are needed. The present experiment was designed to explore what readers are targeting with their return-sweeps. Participants read two short stories by Frank L. Baum while their eye-movements were being recorded. In one story, every line-initial word was highlighted by formatting it in bold, while the other story was presented normally (i.e., without any bolding). The bolding manipulation significantly reduced oculomotor error associated with return-sweeps, as these saccades landed closer to the left margin and were less likely to require corrective saccades compared to the control condition. However, despite this reduction in oculomotor error, the bolding had no influence on local fixation durations or global reading-time measures. Moreover, return-sweep landing sites were not impacted by line-initial word length nor did the effect of bolding interact with the length of the line-initial word, suggesting that readers were not targeting the centre of line-initial words. We discuss the implication of these findings for return-sweep targeting and eye-movement control during reading.

**Electronic supplementary material:**

The online version of this article (10.3758/s13414-019-01742-3) contains supplementary material, which is available to authorized users.

## Introduction

During reading, the eyes alternate between quick movements (known as *saccades*) and short periods of relative stability (known as *fixations*)*.* Most saccades during reading are relatively short and, on average, span about eight characters (Rayner, 1978). However, when reading paragraphs, readers also need to make long saccades that take their eyes from the end of the current line to the beginning of the next. These saccades are known as *return-sweeps* (Rayner, 1998). While return-sweeps are common in everyday reading, little is known about how they are programmed because most eye-movement experiments use single lines of text where return-sweeps are not needed. Here, we explore what readers are targeting with their return-sweeps by manipulating the saliency of the first word on each line.

When readers make intra-line saccades, their eyes tend to land slightly left of the centre of words – known as the *preferred viewing location* (Rayner, 1979). This is thought to occur because readers target the center of words (known as the *optimal viewing position* [OVP]) but undershoot this location due to saccadic range error (McConkie, Kerr, Reddix, & Zola, 1988). The OVP is the fixation location where word processing is optimal (Rayner, 2009). However, fixations that land close to the OVP paradoxically tend to be longer than those landing near the beginning or end of words (Vitu, McConkie, Kerr, & O’Regan, 2001). This counter-intuitive finding is known as the *inverted optimal viewing position* effect (Vitu et al., 2001) and occurs because initial fixations located near word boundaries are more likely to be mislocated and quickly generate a corrective saccade (Nuthmann, Engbert, & Kliegl, 2005).

Return-sweeps differ from intra-line saccades as the eyes traverse a much larger distance, typically around 40–70 characters. Return-sweeps are normally launched from around five characters from the end of the line and land around six characters from the leftmost character of the next line (Hofmeister, Heller, & Radach, 1999; Parker, Slattery, & Kirkby, 2019). However, their landing positions are strongly influenced by line length. With longer lines, the landing position is shifted rightward (Hofmeister et al., 1999). This likely occurs due to saccadic range error – saccades are more likely to undershoot their target when they are launched from further away (McConkie et al., 1988). In this sense, return-sweeps typically have more saccadic range error than intra-line saccades. In fact, return-sweeps often land short of the beginning of a line and are then followed by a corrective saccade to the left (Andriessen & de Voogd, 1973; Hofmeister et al., 1999).

While the basic characteristics of return-sweeps are known, it is not well understood what such saccades are targeting. Because little visual information is obtained from the line below the current fixation (Pollatsek, Raney, Lagasse, & Rayner, 1993), readers likely have a limited, if any, spatial memory of word locations on the next line. Additionally, as return-sweeps are launched from further away, line-initial words will fall outside parafoveal vision. This represents a major difference between return-sweeps and intra-line saccades, which has been hypothesized to prevent parafoveal pre-processing of line-initial words prior to the return-sweep (Parker, Kirkby, & Slattery, 2017). Indeed, on the fixation prior to the return-sweep, line-initial words may even be too far in peripheral vision to be accurately segmented. Therefore, readers may not have access to the word length information needed to target the OVP of line-initial words. Rather, instead of the center of the first word, readers may be targeting an area relative to the leftmost character on the next line.

The present experiment sought to distinguish between these alternatives. If readers target the center of line-initial words, it should be easier to do so when the first word on a line is more salient and its word length information is easier to determine. In this case, the landing position of return-sweeps should shift rightwards with increasing word length because the word’s center will also shift to the right. Alternatively, if readers target the left margin of the line, the landing position of return-sweeps should shift to the left with an increase in salience because the margin will be more prominent. In this experiment, the salience of line-initial words was manipulated by presenting them either normally or in bold.

Research has shown that image statistics, such as contrast, impact fixation locations during scene viewing (Tatler, Baddeley, & Gilchrist, 2005). However, few eye-movement studies of reading have explored the impact of bolding (Hohenstein, Laubrock, & Kliegl, 2010; Perea & Acha, 2009; Reingold & Rayner, 2006; Slattery & Rayner, 2010; see Slattery, 2016). Both Slattery and Rayner (2010) and Reingold and Rayner (2006) included a bolding manipulation but found that it had little to no impact on fixation durations. Perea and Acha (2009) explored the use of an alternating-**bold** condition for word segmentation in the absence of word spaces. While they included an analysis of target-word landing sites, the data are not split by whether the target was printed normally or in bold. Additionally, bold words were used only in a condition without inter-word spaces. Therefore, these data are not useful for predicting the impact of bolding in the current study. However, their finding that readers were less disrupted by the removal of inter-word spaces in the alternating-bold condition indicates that the contrast difference provided by bolding influences word salience. Hohenstein et al. (2010) used bolding in their Experiment 3 to increase the salience of parafoveal words in all conditions and present an analysis of landing sites for the bolded target word. In comparison to Experiments 1 and 2, which used the same stimuli, the relative landing positions (absolute landing position in characters divided by word length) did not differ appreciably between Experiment 1 (mean = .45, SD ~.22), and Experiment 2 (mean = .46, SD ~.22), which did not bold any target words, and Experiment 3 (mean = .45, SD ~.22), which bolded them all. While they present no between-experiment comparisons of landing sites, it seems safe to assume that such small differences relative to the standard deviations indicates that targeting and executing forward intra-line saccades are not influenced by bolding. However, as already mentioned, return-sweeps represent a type of reading saccade distinct from intra-line reading saccades.

We predicted that, if readers are targeting their return-sweeps to the OVP of line-initial words, these return-sweeps should land further to the right as word line initial length increases (i.e., main effect of word length) and that this effect should be more pronounced when the line-initial-word length information is made more salient by bolding these words (i.e., an interaction between line-initial-word bolding and word length). Alternatively, if readers target the left line margin, return-sweeps should land further to the left in the Bold condition regardless of the length of the first word (i.e., a main effect of bolding in the opposite direction predicted by the center targeting hypothesis, but no interaction with word length).

## Method

### Participants

Thirty-two members of the Bournemouth community (20 female) participated for £10 (mean age= 28 years; *SD*= 12.1 years; range: 19–63 years). Participants were English speakers who reported normal or corrected-to-normal vision and no reading disorders. Participants were naïve as to the purpose of the experiment. The study was approved by the Bournemouth University Research Ethics Committee (protocol No. 16769).

### Materials and design

Participants read two illustrated stories from the book “Little Wizard Stories of Oz” by L. Frank Baum (Baum, 2008/1914). The stories were “Little Dorothy and Toto” and “Tiktok and the Nome King.” The lengths of line-initial words varied from 2 to 15 characters (Table 1). The text was divided into 17 and 18 screens for the first and second story, respectively. Each text screen was considered as a separate item in the statistical analyses.^1^ The illustrations appeared on separate screens at the point in which they occurred in the original stories and were not accompanied by any text from the story.

**Table 1.** Word length distribution in letters of the first word on each line in the two stories

Word length	2	3	4	5	6	7	8	9	10	11	12–15
Percent of total	0.63	6.90	19.75	15.36	14.11	13.48	13.48	6.90	5.64	2.19	1.57

**Fig. 1:**
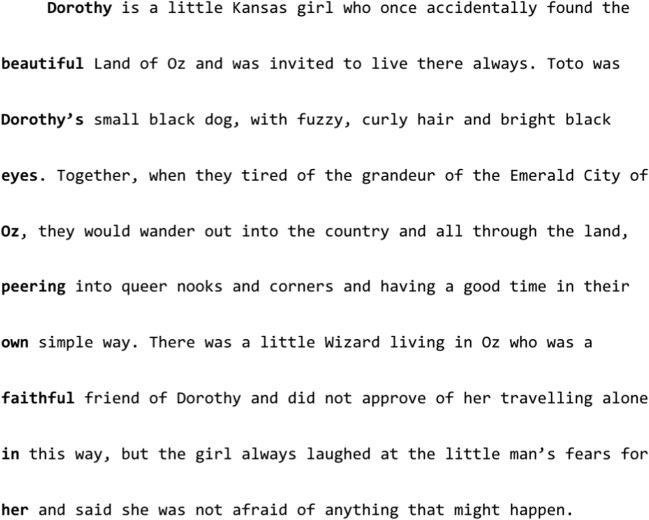
An example page from the story “Little Dorothy and Toto” in the Bold condition. In the Normal condition, each line-initial word was formatted normally (i.e., not in bold)

There were two experimental conditions: (1) *bold-type* in which the first word on each line (and only this first word) was formatted in bold typeface (see Fig. 1); and (2) a *normal-type* control in which the first word on each line was formatted normally (i.e., not in bold). Each story was assigned to one of the two experimental conditions. In the bold-type condition, the bolding on each line remained present for the duration of the whole story. The assignment of conditions and the order of the two stories were counterbalanced with a Latin square design.

### Apparatus

Participants’ eye movements were recorded at 1,000 Hz with an EyeLink 1000 Tower Mount. Viewing was binocular, but only the right eye was recorded.^2^ A chin-and-forehead rest was used to reduce head-movement artefacts. The experiment was programmed in Python 2.7 using the PsychoPy (Peirce, 2007) and PyGaze (Dalmaijer, Mathôt, & Van der Stigchel, 2014).

The texts were presented in a monospaced Consolas 11pt. font and appeared as black text over a white background at the center of a Cambridge Research Systems LCD++ monitor (screen resolution: 1,920 × 1,080 pixels; refresh rate: 100 Hz). The text was double-spaced and aligned to the left. The length of each line of text ranged from five to 91 characters (*M*= 59.9; *SD*= 19.4). Participants sat 80 cm away from the monitor and at this distance each letter subtended approximately 0.30° per visual angle. The experiment was run on a Windows 7 PC.

### Procedure

Participants read the two stories in a 35- to 45-min session. First, participants were calibrated on a 9-point calibration grid. A drift check was presented before each trial and participants were re-calibrated if the error was >.40°. Trials started with a black gaze box, centered at the location of the first letter in the passage. Once a stable fixation on the gaze box was detected, the box disappeared and the text was presented.

Participants were informed that one of the stories would have typographic changes and were instructed to read both stories normally as they would read a book. Participants could move back or forward through the pages by pressing the right and left arrow keys on the keyboard. While they had the opportunity to go back a page, none did so. After each story, participants answered five multiple-choice comprehension questions about its contents by pressing a keyboard button to indicate their answer from four alternatives.

### Data analysis

The experiment had one within-subject factor with two levels: the first word on a line was either formatted in bold type or in a normal type. Our main analysis tested the landing position of return-sweeps on a new line. However, we first explored the impact that our bolding manipulation had on the launch site of return-sweeps. We also examined the probability of making an undersweep-fixation as a function of the experimental condition. Undersweep-fixations occur between a return-sweep and a corrective saccade toward the left margin, and their frequency has been interpreted as a measure of oculomotor error (Parker et al., 2017, 2019). More precisely, an undersweep-fixation is any line-initial fixation that is immediately followed by a leftward saccade regardless of where this fixation lands. Fifty-seven percent of line-initial fixations were undersweeps in the current data, which is similar to other studies of return-sweeps (Hofmiester et al. 1999; Parker et al., 2017, 2019). In comparison, oversweep-fixations that land to the left of the first character on a line were rare (0.97% of line-initial fixations). Finally, we explored whether our bolding manipulation influenced the average time to read a page. Means are presented in Table 2.

**Table 2.** Descriptive statistics for return-sweep launch site, landing position, probability of under-sweep fixation, and page reading time as a function of experimental condition

Experimental condition	Return-sweep launch site (char.)	Return-sweep landing position (char.)	Probability of under-sweep fixation (%)	Page reading time (s)
Normal	57.3 (20.4)	7.2 (4.6)	60.5 (48.9)	23.1 (6.7)
Bold	57.0 (20.6)	6.6 (4.3)	55.6 (49.7)	23.3 (7.2)

*Note:* Standard deviations are shown in parentheses

The data were analyzed with (Generalized) Linear Mixed Models ((G)LMMs) by using the “lme4” package v.1.1-12 (Bates, Machler, Bolker, & Walker, 2014) in R v.3.5.1 (R Core Team, 2018). Participants and items were added as random intercepts in the models (Baayen, Davidson, & Bates, 2008). Additionally, random slopes for the experimental condition were also added for both participants and items (Barr, Levy, Scheepers, & Tily, 2013).^3^ Treatment contrast coding was used for the experimental condition, where Normal text was the baseline. The results were considered as statistically significant if the |*t*| or |*z*| values were ≥1.96.

## Results

The fixation data were manually processed with the EyeDoctor software (Stracuzzi & Kinsey, 2009) to re-align the vertical position of fixations when necessary. Fixations shorter than 80 ms occurring within one character of another fixation were combined with that fixation. Fixations longer than 1,000 ms were removed as outliers (0.03% of all observations). All participants had comprehension accuracy greater than 80%, indicating that they understood the stories. There was no significant difference in comprehension accuracy between the Bold (*M*= 96.2%) and Normal (*M*= 97.5%) conditions, *z*= 0.46.

First, a model was fit predicting the launch position of return-sweeps as a function of experimental condition. The results indicated no difference in return-sweep launch position between the normal and bold conditions (*t*= -0.708), suggesting that the bolding manipulation did not influence where participants launched their return-sweeps from. The experimental manipulation also had no influence on fixation durations of line-initial words, further suggesting that bolding did not affect the lexical processing of these words (see the Supplementary Material).

**Fig. 2:**
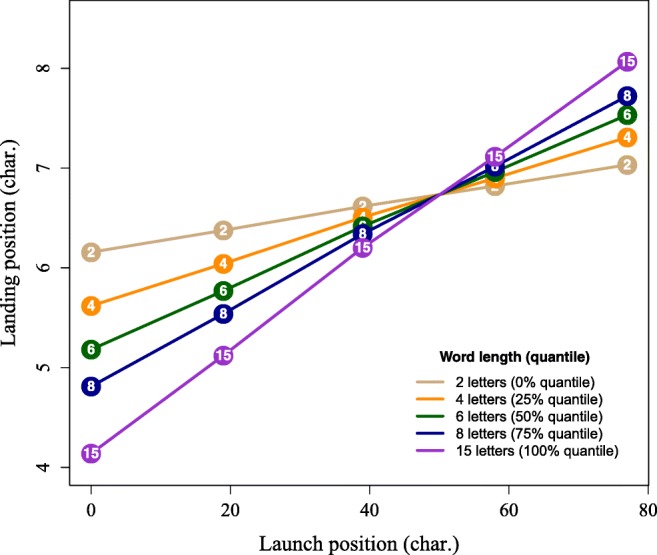
Illustration of the landing position interaction between launch position and line-initial word length in Table 2. The word length numbers in the graph correspond to the quantiles of the word length probability distribution. Means were extracted with the “effects” R-package v.4.0-3 (Fox & Hong, 2009)

The return-sweep landing position results are presented in Table 3. There was a main effect of experimental condition, indicating that participants landed, on average, 0.54 characters closer to the beginning of the new line in the bold compared to the normal condition. Critically, however, the interaction between bold and length of the first word was not significant. Additionally, there was a main effect of launch site, as participants landed farther from the beginning of a new line when they also launched from further away (i.e., closer to the end of the previous line). Furthermore, there was a significant interaction between launch site and line-initial word length (see Fig. 2). This was due to participants landing further away from the beginning of the new line with increasing length of the first word, but only when the launch position was distant (approx. >50 characters); when the launch site was closer to the beginning of the new line, the reverse trend was observed and the saccade landed closer to the start of the new line with increasing length of the first word.

**Table 3.** Linear mixed model (LMM) results for landing position in characters relative to the start of the new line as a function of experimental condition, launch site, and length of the first word on the line

Effect	b	SE	t
Intercept	7.175	0.42	**17.088**
Bold	-0.543	0.14	**-3.881**
Launch	0.528	0.06	**8.799**
W1 Length	0.013	0.058	0.214
Bold × Launch	-0.11	0.083	-1.322
Bold × W1 Length	0.066	0.081	0.805
Launch × W1 Length	0.14	0.07	**2.017**
Bold × Launch × W1 Length	-0.026	0.096	-0.271

*Note*: Statistically significant *t*-values are formatted in **bold**

*Bold* Experimental condition effect (bold vs. normal), *Launch:* launch position of the return-sweep saccade in characters (centred at 0), *W1 Length* length of the first word on a line in characters (centred at 0)

The GLMM results of under-sweep probability are presented in Table 4. There was a main effect of experimental condition, indicating that undersweep-fixations were less likely in the bold compared to the normal condition. Additionally, there was a main effect of launch position, which was due to a greater probability of making an undersweep-fixation with increasing distance between the launch site and the beginning of the new line. Interestingly, while the main effect of experimental condition was significant, the interaction with launch site was not. This suggests that the bold condition reduced the probability of making an under-sweep fixation regardless of the location from which participants launched their return-sweep.

**Table 4.** Generalized linear mixed model (GLMM) results of under-sweep probability in the experiment

Effect	b	SE	z
(Intercept)	0.535	0.174	**3.076**
Bold	-0.25	0.062	**-4.026**
Launch	0.673	0.037	**18.122**
Bold × Launch	0.02	0.052	0.382

*Note*: Statistically significant *z*-values are formatted in **bold**

*Bold* Experimental condition effect (bold vs. normal), *Launch* launch position of the return-sweep saccade (centred at 0)

The analysis of return-sweep landing positions indicates that the bolding manipulation resulted in readers landing closer to the start of lines. Moreover, the reduction in undersweep-fixation probability suggests that return-sweeps in the bold condition landed closer to their intended target. Given these results, we explored whether this decrease in oculomotor error resulted in faster, more efficient reading of pages in the bold condition. We fit an LMM to the log-transformed page reading time (in seconds; Table 5). The average page reading time was 23.1 s in the normal condition and 23.3 s in the bold condition – a difference that was not significant. Therefore, while the bolding reduced oculomotor error, it did not improve reading efficiency.

**Table 5.** Linear mixed model (LMM) results of page reading time in the experiment

Effect	b	SE	z
(Intercept)	3.102	0.044	**70.926**
Bold	0.001	0.020	0.026

*Note*: Statistically significant *z*-values are formatted in **bold**

*Bold* Experimental condition effect (Bold vs. Normal)

## Discussion

The present experiment manipulated the saliency of line-initial words to test what readers are targeting with their return-sweeps. If readers are targetting their return-sweeps to the centers of words similar to the targetting of intra-line reading saccades, then the landing sites of return-sweeps should shift to the right as the length of line-initial word grows. Moreover, this word-center targetting hypothesis predicts that a word length effect should be larger when the line-initial word length information is made more salient with bolding. However, consistent with our alternative hypothesis, the results showed that return-sweeps in the salient bold condition landed closer to the left margin regardless of the length of the first word. This bolding effect on return-sweeps when compared to the lack of influence that bolding had on landing sites in Hohenstein et al. (2010), is further evidence that return-sweeps are distinct from forward intra-line saccades. Additionally, the bold condition reduced the probability of making a leftward corrective saccade after the return-sweep. The length of line-initial words had neither a main effect nor an interaction with bolding and therefore did not influence return-sweep landing sites. So, there was no support for the prediction that readers target the OVP of line-initial words. Rather, the present data suggest that readers target some area relative to the left margin of the line.

One unexpected finding was the interaction between launch position and line-initial word length on landing positions. Landing positions shifted closer to the left margin with increasing word length, but only for closer launch sites. This may occur if readers are using the empty space after the first word to help them segment the text and locate the left margin. With longer line-initial words, the space shifts closer to the launch position (and thus towards foveal vision). When this launch position is closer to the start of the line, it may be a reliable targeting cue. However, this speculation needs to be tested in future research.

Interestingly, while the bolding manipulation reduced the oculomotor error associated with return-sweeps, it did not improve overall reading efficiency. This suggests that adult readers have learned to make the most out of the information gleaned during the undersweep-fixations that intervene between a return-sweep and a corrective saccade. This is consistent with recent research showing that readers obtain significant preview benefit of line-initial words during undersweep-fixations (Parker & Slattery, submitted). Moreover, Slattery and Parker (accepted) reported that readers pre-attentively process the words at the locations of undersweep-fixations, which benefits subsequent reading of the line. So, when skilled adult readers make an undersweep fixation, they obtain information from nearby words that aids their recognition. Reducing undersweep-fixations will not necessarily increase reading speed because this information still needs to be obtained on subsequent fixations. However, it is unclear if such benefits would exist for young developing readers or dyslexic readers. It may be the case that such readers would be more hindered by the inherent oculomotor error of return-sweeps and therefore would show increased reading efficiency when line-initial words are in bold.

Current models of eye-movement control during reading (e.g., Engbert, Nuthmann, Richter, & Kliegl, 2005; Reichle, Warren, & McConnell, 2009; Snell, van Leipsig, Grainger, & Meeter, 2018) have used only single-line reading where return-sweeps are absent. However, a complete model of reading will inevitably need to account for return-sweeps. The present results suggest that return-sweeps are targetted differently from intra-line saccades. Because exisiting models assume that saccades always target the OVP of words (Engbert et al., 2005; Reichle et al., 2009), a separate saccade targeting mechanism may need to be implemented for return-sweeps. Additionally, simulating return-sweeps may require changing the amount of random and systematic saccadic range error associated with such saccades due to the greater distance that the eyes travel. SWIFT (Engbert et al., 2005) already uses different occulomotor error values for different types of saccades (e.g., forward, regressive). Therefore, future models may adopt a similar approach where return-sweep saccades use different occulomotor error values compared to intra-line saccades. The above-mentioned dissociation between reading efficiency and return-sweep error will also be important for computational models to capture. The chief difference between EZ-Reader and SWIFT is whether lexical processing is occuring serially or in parallel, respectively. For SWIFT, since lexical processing is happening across multiple words around fixation, there should be little cost to overall reading efficiency when fixations are mislocated due to return-sweep error. However, EZ-Reader has a strict serial assumption where lexical processing is concerned. Therefore, the model may have more difficulty explaining how reading efficiency is not increased under conditions where return-sweep error is reduced. However, Slattery and Parker (accepted) suggest that EZ-Reader’s pre-attentive visual processing stage (V), which operates in parallel, may be capable of explaining the dissociation based on processing of abstract letter identities.

In summary, the current study helped rule out the possibility that readers target the OVP of line-initial words with their return-sweeps. Furthemore, it adds to the growing body of evidence indicating that skilled readers have learned to make the most of the visual information obtained at unintended fixation locations (Parker & Slattery, submitted; Slattery & Parker, accepted). However, more research is required to understand the complexities of return-sweeps and their role in linguistic processing.

## Electronic supplementary material

ESM 1 (DOCX 250 kb)

## Footnotes

1 This was done because return-sweeps are a global-reading phenomenon that spans two lines of text and they are also influenced by the properties of line from which they are launched (e.g., Hofmeister, 1998). Additionally, a single page is the smallest meaningful unit that would make it possible to generalize the results to different types of text that participants could be reading (see Clark, 1973; Coleman, 1964).

2 The left eye was recorded for four participants as a result of tracking problems with their right eye due to glasses or contact lenses.

3 The random slope for items was removed for the comprehension accuracy and under-sweep probability models due to convergence failure.
