# Supplementary material for: An eye-movement exploration into return-sweep targeting during reading
Source: Atten Percept Psychophys. 2019 Jun 4;81(5):1197–203. doi: 10.3758/s13414-019-01742-3 (PMC6647890; doi:10.3758/s13414-019-01742-3)
Supplement: Supplementary file 1 — (DOCX 250 kb) [file 13414_2019_1742_MOESM1_ESM.docx]

**Supplementary Materials**

Here we present an analysis that tested whether the Bold text condition affected the durations of the four types of fixations in the experiment: 1) *intra-line fixations* (i.e., fixations that are not immediately preceded or followed by a return-sweep), *line-final fixations* (i.e., the fixation immediately prior to a return-sweep to the next line), *under-sweep fixations* (i.e., fixations immediately after a return-sweep that land short of the start of the line and are then followed by a leftward corrective saccade), and *accurate-sweep* *fixations* (i.e., fixations immediately after a return-sweep that land close enough to the start of a new line that they are followed by a rightward saccade).

The data were analysed with Linear Mixed Models (LMMs) by using the “lme4” package v.1.1-12 (Bates, Machler, Bolker, & Walker, 2014) in the R statistical software v.3.5.1 (R Core Team, 2018). Fixation durations were log-transformed in all models. Participants and items were added as random intercepts in the models (Baayen, Davidson, & Bates, 2008). Additionally, random intercepts for experimental condition were also added for both participants and items (Barr, Levy, Scheepers, & Tily, 2013). Treatment contrast coding was used for the experimental condition, where Normal text was the baseline. Treatment contrast coding was also used for the fixation type variable, with intra-line fixations as the baseline. The results were considered as statistically significant if the |*t*| or |*z*| values were ≥ 1.96.

The distribution of fixation durations for the four fixation types is shown in Figure S1. Line-final (*b*= -0.158, *SE*= 0.006, *t*= -26.21) and under-sweep fixations (*b*= -0.286, *SE*= 0.007, *t*= -37.45) were significantly shorter than intra-line fixations. In contrast, accurate-sweep fixations were significantly longer than intra-line fixations (*b*= 0.174, *SE*= 0.009, *t*= 18.97). There was no main effect of experimental condition (*b*= -0.006, *SE*= 0.007, *t*= -0.845) or any interactions between experimental condition and fixation type (all |*t|*s ≤ 0.617). Therefore, participants spent less time fixating the end of the line immediately before a return-sweep and when they undershoot the start of the next line. In contrast, participants fixated longer at the beginning of a new line after making an accurate return-sweep. Critically, however, the bold text condition had no influence on the duration of any of the four fixation types.


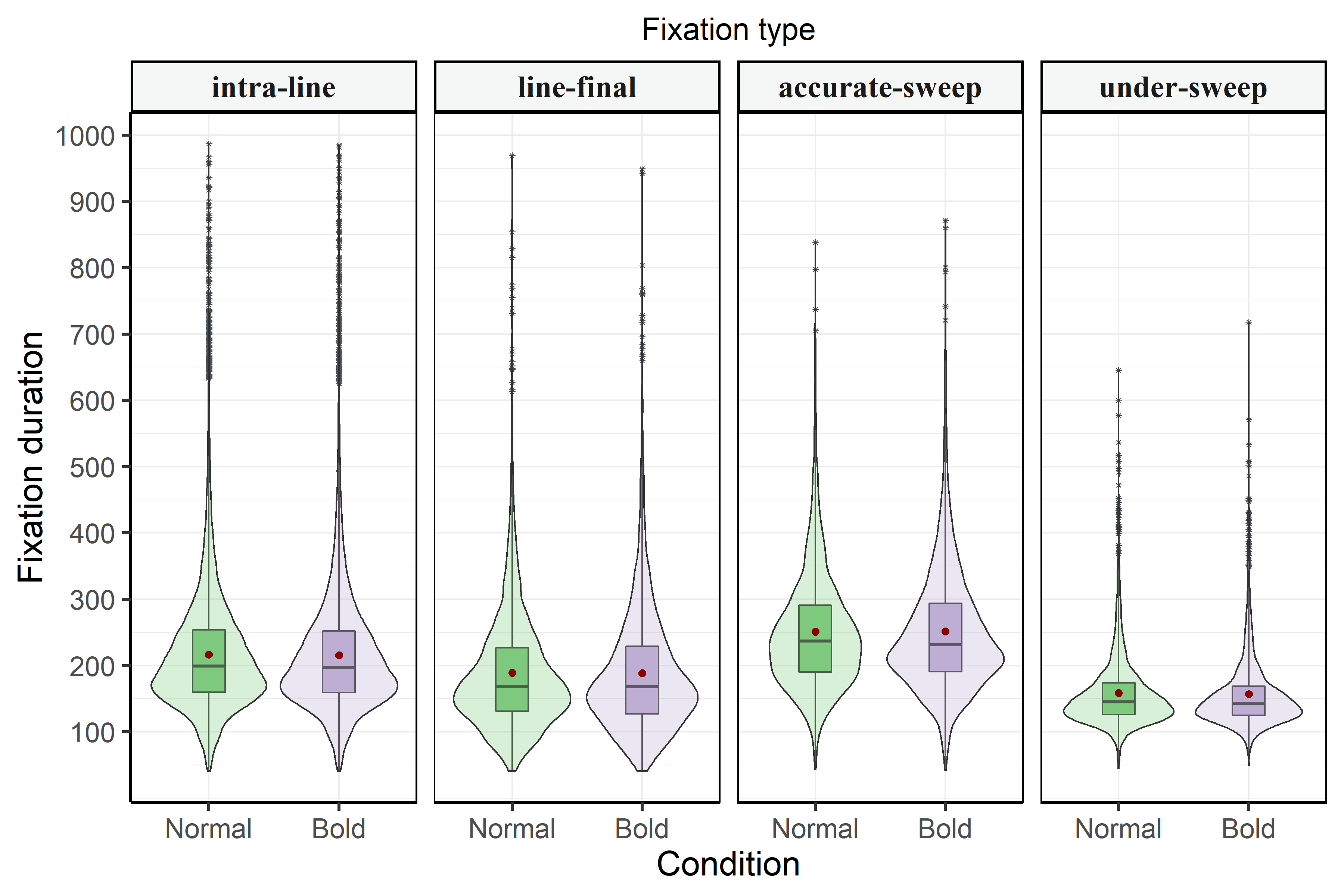


*Figure 2*. Probability density and box plots of the four fixation types as a function of experimental condition (Normal vs. Bold). Horizontal black lines on the boxplots indicate the median and dark red circles indicate the mean.

A separate analysis was done to test if the bolding manipulation affected fixation durations on line-initial words (see Table S1). In cases where there was an undersweep-fixation beyond the line-initial word, we calculated the gaze duration for the line-initial word ignoring the undersweep (see Parker & Slattery, submitted; Parker, Slattery, & Kirkby, 2019; Slattery & Parker, submitted). The standard lexical frequency and word length effects were significant for both gaze duration and total viewing time, indicating that fixation durations became longer with decreasing lexical frequency and increasing word length. However, there was no main effect of the bold condition or any interactions with lexical frequency or word length. This suggests that the experimental manipulation had no influence on the lexical processing of line-initial words.

Table S1

*LMM Results for Fixation Duration Measures on Line-initial Words in the Text*

| Effect | Gaze duration | | | Total Viewing Time | | |
| --- | --- | --- | --- | --- | --- | --- |
|  | b | SE | t | b | SE | t |
| Intercept | 5.753 | 0.047 | **122.07** | 5.839 | 0.05 | **117.55** |
| Bold | -0.031 | 0.052 | -0.586 | -0.012 | 0.058 | -0.214 |
| Freq | -0.025 | 0.003 | **-7.689** | -0.029 | 0.004 | **-8.101** |
| WordLen | 0.093 | 0.026 | **3.57** | 0.1 | 0.029 | **3.491** |
| Bold x Freq | 0.001 | 0.005 | 0.301 | 0.001 | 0.005 | 0.197 |
| Bold x WordLen | 0.054 | 0.036 | 1.489 | 0.053 | 0.04 | 1.328 |
| Freq x WordLen | -0.004 | 0.003 | -1.565 | -0.005 | 0.003 | -1.805 |
| Bold x Freq x WordLen | -0.004 | 0.004 | -1.184 | -0.004 | 0.004 | -0.911 |

*Note*: Statistically significant *t*-values are formatted in bold. Freq: lexical frequency (log transformed). WordLen: word length (centred at 0). Because of the literary style of the stories, 10 % of the line-initial words did not have lexical frequency entries in the SUBTLEX-UK database (Van Heuven, Mandera, Keuleers, & Brysbaert, 2014). These words were excluded from the analyses.
